# Unraveling the cartography of the cancer ecosystem

**DOI:** 10.1186/s13059-021-02310-5

**Published:** 2021-03-24

**Authors:** Roy Rabbie, Doreen Lau, Richard M. White, David J. Adams

**Affiliations:** 1 Cambridge Cancer Centre, Cambridge University Hospitals NHS Foundation Trust, Cambridge, UK; 2 Experimental Cancer Genetics, The Wellcome Sanger Institute, Hinxton, Cambridge, Cambridgeshire UK; 3 Cancer Research UK Cambridge Centre, University of Cambridge, Cambridge, CB2 0RE UK; 4 Department of Radiology, School of Clinical Medicine, University of Cambridge, Box 218, Cambridge Biomedical Campus, Cambridge, UK; 5 Department of Cancer Biology & Genetics and Department of Medicine, Memorial Sloan Kettering Cancer Center, New York, NY USA

## Background

Tumors are complex multifaceted ecosystems composed of malignant cells surrounded by a heterogeneous mixture of cell types. During oncogenesis, different populations of cancer cells interact with the microenvironment, contributing to evasion of the immune system and metastatic progression. One of the major goals for translational cancer research is to develop new technologies capable of unraveling this complexity across heterogeneous microenvironments. Bulk genomic and transcriptomic studies have uncovered major molecular insights and have helped in the design of patient-specific targeted therapeutics. Quantification of gene expression from bulk-sequencing approaches, however, only represents the average expression profiles of the constituent cells and is influenced by the particular transcriptional profiles, as well as the abundance of a multitude of different cell types and states within each sample. This becomes particularly relevant when considering the detection limits that might preclude the identification of low-level subclones. The development of technologies based on sequencing individual cells over the past decade has been astonishing. Notably, spatial molecular analysis of RNA and protein now place cellular biology at the center of cancer biology and may be used to dissect interactions across tumor microenvironments.

## Morphologic examination

Spatial mapping of tumor, immune and stromal cells within their microenvironment has long been documented by histopathologic observations. However, specimens may contain hundreds or thousands of cells that can display extensive intratumoral morphological heterogeneity. For example, in many malignant tumors, it is common to find well-differentiated regions adjacent to poorly or moderately differentiated regions, or more than one morphological pattern that, if not taken into account, can lead to an inaccurate or even incorrect diagnosis [1]. Although metastasis remains the primary cause of mortality, decisions made during a patient’s treatment are often based on the histopathologic or molecular analyses of biopsy specimens available at the site or time of initial diagnosis. Studies on the divergent evolution of metastatic tumors, however, suggest that the biopsy of a primary tumor may not necessarily be reflective of secondary deposits, particularly following the selective pressures of therapies [2]. Microenvironmental factors such as inflammation, angiogenesis and hypoxia can also induce changes in gene and protein expression and downstream responses to anticancer therapeutics. In addition, core biopsies often only reflect a snapshot of the whole tumor and are therefore unlikely to be fully informative of the complete clonal composition of tumors.

**Fig. 1:**
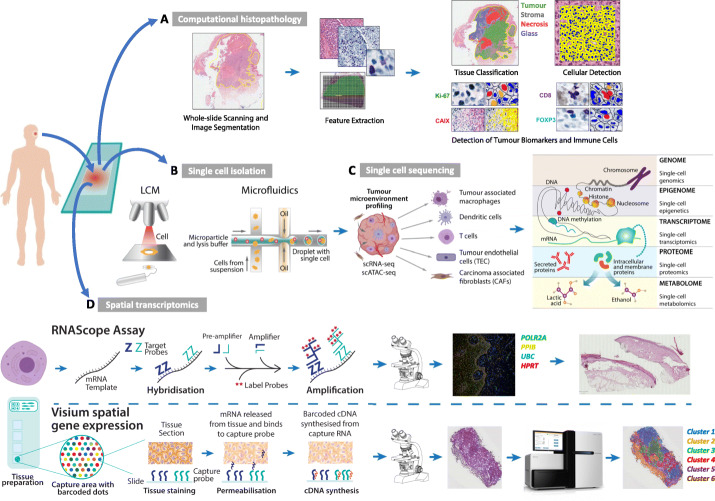
Profiling the cartography of the cancer ecosystem. (A) Hematoxylin and eosin (H&E) and chromogen-based immunohistochemical stained images of a primary cutaneous melanoma, showing the result of a machine learning algorithm capable of detecting image features that distinguish tumor from stromal and necrotic regions, and quantify the number of cancer cells expressing key biomarkers, as well as counts of tumor-infiltrating immune cells. (B) Single-cell isolation approaches include high-throughput microfluidic technologies and laser capture microdissection (LCM), adapted from [3]. (C) Technologies are now able to dissect single tumor cells at previously unattainable resolutions exemplified by approaches to analyze the genome, transcriptome and epigenome, adapted from [4]. (D) Gene expression patterns can also be spatially resolved across microenvironmental conditions. Schematic workflow of spatial transcriptomic analyses using fluorescence and Visium spatial technologies. Schematic of the RNAscope assay. After the tissue is permabilized to allow probe access, target RNA-specific oligonucleotide probes (Z) are hybridized to the RNA target template. Once adjacent target (Z) probe-pairs hybridize to the RNA template, the preamplifier binds to the Z probe, followed by the binding multiple amplification molecules. Each probe is conjugated to a different fluorophore which are detected using a fluorescent microscope. Workflow adapted from [5]. Shown H&E of a primary acral melanoma and the associated 4-plex RNAscope smFISH image, staining for 4 positive control genes. In the 10X Visium platform, tissue samples are sectioned and aligned onto the capture areas of the gene expression slides. Once the tissue is permeabilized, mRNA are hybridized to the capture probes and converted into a spatially barcoded cDNA library by reverse transcription. Sequencing the library then allows the cDNA barcodes to associate to a slide coordinate within the original tissue, mapping the gene expression profile to its spatial localization. Original image adapted with permission from 10x Genomics graphic. Shown H&E of a T3 primary lung adenocarcinoma and the associated spatially resolved gene expression plot, showing 6 mRNA clusters

In recent years, remarkable progress has been made in the objective assessment of the cellular context of an entire slide captured on digitalized histological sections. Modern digital image acquisition and quantification algorithms, which integrate biophysical parameters to capture spatial variation in tumor architecture, enable semi-automated computational identification and classification of various cell types and regions (Fig. 1A). Deep learning approaches in particular can identify recurring patterns in information-rich histopathologic images that can then be used to infer molecular features and predict clinical outcomes [6].

It is important however to recognize that unlike in vivo molecular imaging approaches, such as positron emission tomography (PET) and magnetic resonance imaging (MRI), histological slides represent a two-dimensional picture of a three-dimensional tumor captured at a point in time and are usually limited to just 5–10-μm sample tissue sections. In addition, it is well recognized that image analyses are very sensitive to sample quality as well as the undesirable effects resulting from specimen processing that could adversely affect their interpretation. Therefore, it is imperative that methods are developed to accommodate the significant variation seen in histological specimens.

## The single-cell revolution

It comes as no surprise that single-cell genomic, transcriptomic and epigenomic sequencing approaches have advanced rapidly over the past decade. The first and most widely available technology at the forefront of single-cell interrogation in a high-throughput manner is single-cell RNA sequencing (scRNA-seq). These methods measure the transcriptional output of cells directly from tumor samples and have now become the dominant technology for the identification of novel cell types and the study of stochastic gene expression.

The first step of all scRNA-seq workflows is the isolation of viable single cells from complex multicellular solid tissues (Fig. 1 (B)). Defining the taxonomy of single cells has historically been achieved using fluorescence-activated cell sorting (FACS) or flow cytometry approaches, whereby cells are tagged with a specific antibody recognizing distinct cellular populations. Although these widely used approaches provide high cellular resolution, they only allow a limited number of molecular markers to be assayed per cell. More recently, laser-capture microdissection using a laser to microscopically isolate single cells based on cellular morphology within a spatially preserved fixed or frozen tissue section has been used [7] (Fig. 1 (B)). Promising advances in the use of microfluidic technologies have also entered mainstream use [8]. In microfluidics, small volumes of fluids are sequentially manipulated into micron-diameter channels to isolate single cells for culture and/or sequencing, allowing target cells to then be separated based on their physical characteristics (e.g., diameter, surface antigen) (Fig. 1 (B)). In addition to their high-throughput scale, microfluidic devices and droplet-based microfluidics also improve the sensitivity of single-cell analyses by confining and concentrating the reaction volume. Approaches such as Drop-seq are unparalleled in the large numbers of cells that can be profiled (> 10,000). When combined with oligo-tagged antibodies in cell hashing methods, it is possible to trace each individual cell back to its original sample [9], thereby identifying genetically identical samples subjected to diverse perturbations in a range of experimental models, e.g., using scRNA-Seq to assess the sensitivity of primary tumor cells to drug treatments across different experimental conditions.

However, despite a dramatic increase in the number of studies, single-cell analysis remains a new field and substantial limitations and technical challenges still exist. Single-cell transcriptomic studies in cancer are currently often complicated by elaborate study designs including samples collected at different time points or from patients with different outcomes (e.g., responders and non-responders). Although this might reveal important transcriptional pathways in particular disease states, the clustering of cells by sample or batch (rather than cell type or state) may limit the identification of shared cell types required for rational downstream biological interpretation. In addition, disparate studies of smaller cell pools could lead to false-negative findings of relevance to only a subset of cells and amplify confounding factors relating to a specific study/sequencing modality. Further, scRNA-seq provides only a partial sampling of the transcriptome, with a bias towards more highly expressed genes. Low coverage can result in extreme data sparsity and it can be difficult for example to distinguish rare cell types from technical artifacts using traditional clustering algorithms; this may be particularly pertinent to the higher throughput scRNA-seq technologies.

Bulk tissues consist of millions of cells, but contemporary studies often only sequence thousands to tens of thousands of single cells because of technological limitations and high equipment and sequencing costs. Tissue specimens may be contaminated with blood or other tissues. As such, not all subsets identified in single-cell data may represent the distribution of cells in the entire tissue and it therefore may not be suited for the profiling of sub-optimally preserved or handled clinical specimens. A further limitation in the application of scRNA-seq to solid tumor samples is the requirement for complex dissociation protocols to obtain viable, individualized fresh cells.

At the DNA level, single-cell genomic sequencing (scDNA-seq) technologies have been used to reconstruct cell lineages, track subclones (subpopulations of cells with distinct genotypes), and infer evolutionary trajectories. However, allelic dropouts (i.e., preferential amplification and sequencing of only one allele of a particular gene) and non-uniform genome coverage may hinder the accurate detection of single nucleotide variants (SNVs). This may not apply to transcriptome sequencing approaches, whereby lower sequencing depth can still provide robust information about cell identity. It is also clear that metastatic propensity may not be exclusively encoded within nucleotide sequences, and recent progress in methods to probe the epigenetic regulation of gene expression at single-cell level (including chromatin accessibility via ATAC-seq [10], DNA methylation [11] and others) has been made (Fig. 1 (C)). The considerable technical challenges associated with either amplifying bisulfite-treated single-cell DNA (scBS-seq) or using single-cell chromatin immune-precipitation approaches to sequence chromatin binding proteins (ChIP-seq) have thus far slowed epigenomic profiling from occupying center stage in cancer studies. Of note, there have been marked advances in “multiomic” approaches at the single-cell level, which allows for various combinations of RNA-seq, ATAC-seq, Hi-C, and methylation-sequencing [12–15] which should enable a better integration of epigenetics, transcriptomics and genetics over the next few years.

Despite these challenges, if these protocols are optimized for all applications and become more cost effective and broadly implemented, it is easy to anticipate a significant increase in the application of single-cell technologies within clinical research. For example, strategies for genome sequencing single nuclei obtained from formalin-fixed paraffin-embedded (FFPE) material [16] (how the majority of cancer tissue is processed and stored) will prove particularly relevant to cancer trials. We anticipate that while single-cell datasets may not supplant bulk genomic and transcriptomic tumor profiling (from large consortia such as The Cancer Genome Atlas/The International Cancer Genome Consortium), they will undoubtedly be increasingly integrated with existing and new profiling methods as well as experimental approaches to further dissect intra-tumoral heterogeneity.

## Multicellular spatial modeling of the tumor microenvironment

It is increasingly clear that understanding genetic or epigenetic alterations within tumor cells only represents part of the picture and tumor progression depends on the spatial interactions between tumors and the multicellular tumor microenvironment (TME). For example, it is likely that the transcriptional state of a cancer cell is strongly influenced by its immediate neighbors via physical contact, secreted factors, or metabolite exchange. This implies that to better understand various cancer cell states, we must have a better understanding of the “architecture” of the tumor/TME pairing. Notably, most of the aforementioned multidimensional single-cell techniques first rely on tumor dissociation to obtain cellular suspensions. Such approaches may therefore alter the expression of specific cell surface markers and do not conserve information on the topological organization of cells within particular tissues [17]. Coupling scRNA technologies with optical imaging methods may provide higher spatiotemporal resolution.

There have been major advances in techniques to analyze gene expression in intact tissues that preserve the architecture. Single-molecule fluorescent in situ hybridization (smFISH) employs multiple single-stranded, fluorescently labeled, short DNA probes that hybridize to target cellular mRNAs in fixed cells, allowing for both quantification and localization of RNAs in individual cells. Over the past few years massively paralleled approaches to smFISH have been developed including sequential barcoded fluorescence in situ hybridization (seqFISH) and multiplexed error-robust fluorescence in situ hybridization (merFISH). In these approaches, multiple cycles of hybridization are used to detect and image hundreds to thousands of different mRNA species simultaneously and at high spatial resolution (sub-diffraction limit) [18]. An alternative to the extraction of single cells from tissues is the capture of transcripts directly on intact tissue sections [19]. The commercial RNAscope technology uses a novel probe design strategy and a series of hybridization and amplification steps to reduce the background noise and achieve single molecule visualization in individual cells (Fig. 1 (D)). In the 10X Visium spatial gene expression assay, a tissue section is placed on a microscopic glass slide containing thousands of capture spots that each contain spatially barcoded capture oligonucleotides; these imprint the spatial localization of the mRNA. The mRNAs are then converted to spatially barcoded cDNAs which are then extracted for sequencing. Superimposing the barcoded reads back onto the tissue image provides a spatially resolved transcriptome (Fig. 1 (D)) [5].

However, one limitation with array-based methods is that the resolution is limited to the size of each spot on the array (currently ~ 55 μM), such that each spot can contain more than one cell. Therefore, the observed expression profile at one spot could arise from a mixture of transcripts originating from different cell types. This can be improved by combining it with single-cell and computational techniques such as multimodal intersection analysis (MIA), which maps the spatial enrichment of specific cellular subpopulations and functional states within a heterogenous sample [20]. Similar more recent computational approaches markedly increase the resolution of each spot without the need for additional single-cell sequencing [21, 22]. Perhaps one of the most exciting avenues in this field is the advent of in situ sequencing rather than array-based approaches. One elegant example of this is expansion sequencing (ExSeq). This approach combines simultaneous tissue expansion microscopy with direct in situ genome sequencing and is able to achieve untargeted sequencing of cells within intact biological samples including the mouse brain and metastatic breast cancer samples [23]. The high resolution of this technique also allows for subcellular localization of transcripts such as to the dendrites of individual neurons and we anticipate this technology extending to a range of cell types and intact tissues. Finally, although more nascent, attention is now also being paid to spatially resolved chromatin profiling with methods such as sciMAP-ATAC and these approaches are likely grow over the next few years [24]. It is important to point out that all of these methods remain technically demanding, requiring advanced equipment and image analyses workflows, as well as a large data storage capacity. They also share some limitations with scRNA-seq, including a high dropout rate and limited cellular resolution.

Modern technological advances have made use of mass-tag labeled antibodies, which have greatly expanded the number of markers that can be applied to tissue slides. These ion-based mass cytometry platforms first stain the cells with antibodies conjugated to heavy metal isotopes, which can then be detected by time-of-flight secondary ion mass spectrometry (SIMS) and quantified by multiplexed ion beam imaging (MIBI) [25]. This approach enables multiplexed antibody labeling (potentially up to 100 markers per cell) and quantification of protein abundance at high subcellular resolution. These capabilities mean MIBI is uniquely suited to profile the spatial composition of immune cell subsets within the complex TME, e.g., a study in triple-negative breast cancer (from archival FFPE tissue sections) reported differential enrichment of immunoregulatory proteins across specific immune cell subtypes and patients, and showed that a *compartmentalized* histology, in which the immune cells were spatially segregated from the tumor cells, conferred a survival advantage over samples were tumor and immune cells were mixed [26].

Heterogeneity in the spatial distribution of metabolites within the tumor microenvironment (including endogenous, immunosuppressive, and drug metabolites) also plays a crucial role in influencing gene expression and determining the cellular response to therapies [27]. Metabolic profiling of native tissues in a spatially resolved manner is more challenging than spatial RNA profiling, as amplification of the metabolite signal is limited and labeling toolkits are lacking. In addition, the detection of metabolites and their chemical structures at subcellular resolution is too complex to be deconvolve from a spatial resolution of 5–20 μm provided by conventional mass spectrometry imaging. However, advanced instrumentation approaches such as the atmospheric pressure matrix-assisted laser desorption/ionization (MALDI) which enables imaging of metabolites, lipids, and peptides in single cells at 1.4 μm resolution [28], and the NanoSIMS which allows for the spatial mapping of metabolites, lipids, and carbohydrates at 50–100 nm resolution [29], may overcome some of these limitations.

Moving forwards, it will be important to integrate comprehensive clinical and experimental data from specific cell populations with data from these multidimensional datasets. This integrated multi-omics approach would provide important insights into the complexity of the cancer ecosystem and interactions of the different components of the tumor microenvironment. One of the most pressing challenges in this field is both the analysis and visualization of these highly complex, multidimensional datasets. This problem becomes especially acute when single-cell spatially resolved methods become mainstream, since they will then have to take into account tissue architecture in 3D [30]. It is likely that understanding these data will require the application of machine learning-based methods, which can augment what histology and molecular profiling individually can reveal [31]. To translate these findings to the clinic will ultimately necessitate effective collaboration across multi-disciplinary research teams composed of molecular biologists, computational biologists, data visualization teams and clinicians.

It is our belief that the continued open exchange and sharing of data, expertise and technology (as demonstrated by recent large consortia [32, 33]) will prove critical to drive innovation. Studies conducted on an international scale, ensuring adequate representation of all major world populations, with open-source exchange and standardization of performance metrics, will accrue the most durable benefit.
